# Same-Day Discharge Following Elective Transcatheter Edge-to-Edge Repair

**DOI:** 10.1016/j.jscai.2024.101357

**Published:** 2024-02-28

**Authors:** Harish Sharma, Mark Zorman, Cara Barnes, James D. Newton, Thomas J. Cahill, Sam Dawkins

**Affiliations:** a Oxford Heart Centre, Oxford University Hospitals, Oxford, United Kingdom; b Department of Cardiology, Queen Elizabeth Hospital, Birmingham, United Kingdom

## Introduction

Transcatheter edge-to-edge repair (TEER) has become an established treatment for mitral and tricuspid regurgitation for patients at increased surgical risk. Despite the high rates of procedural success^1^^,^^2^ and transfemoral venous access, elective patients are rarely discharged on the day of the procedure. Recent studies have reported post-TEER median hospital stay of 1 day (IQR, 1-2 days^2^, and 1-4 days^3^). Same-day discharge (SDD) offers potential advantages to patients and health care systems, including early mobilization, facilitated recovery in the home environment, reduced risk of nosocomial infection, and improved cost efficiency. There are few studies on the feasibility and safety of SDD after TEER, however, particularly in patients treated by tricuspid TEER.^4^^,^^5^ The aim of this study was to evaluate outcomes after SDD in patients who underwent TEER.

## Methods

**Figure 1:**
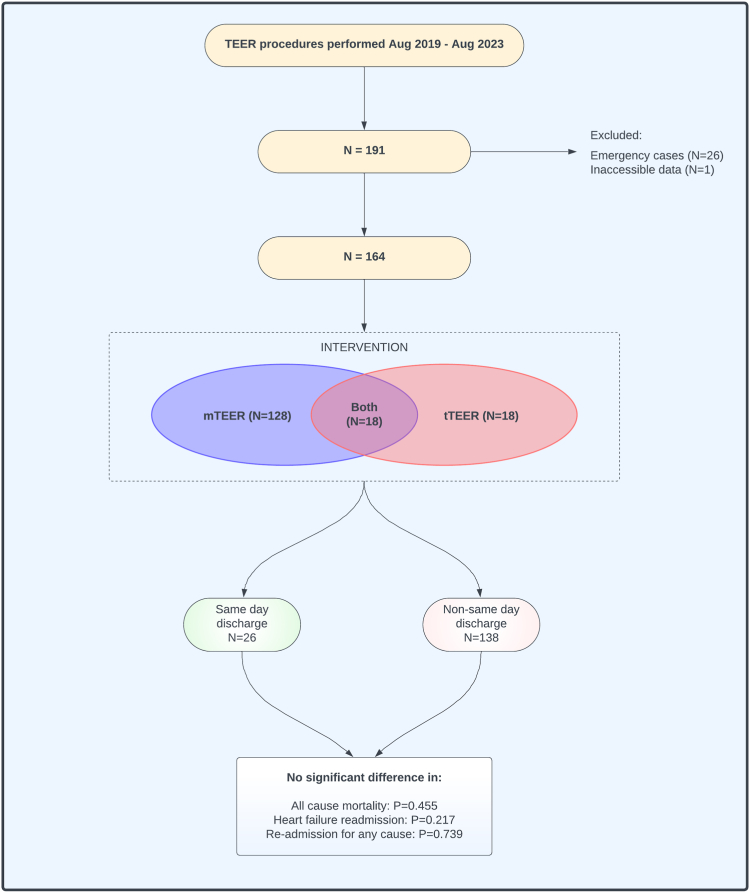
**Summary of the study design and outcomes.** mTEER, mitral transcatheter edge-to-edge repair; tTEER, tricuspid transcatheter edge-to-edge repair.

All TEER procedures performed between August 1, 2019, and August 8, 2023, at the Oxford Heart Center (Oxford, United Kingdom) were retrospectively identified (n = 191). Nonelective cases (n = 26) and those with inaccessible data (n = 1) were excluded. A total of 164 patients were included who underwent TEER of the mitral valve (mTEER; n = 128), tricuspid valve (tTEER; n = 18) or both valves in combination (n = 18) (Figure 1). Patients were considered for SDD if they satisfied the following criteria: 4 hours of postprocedure observation with no significant hematoma formation, an electrocardiogram demonstrating no new arrhythmias, focused bedside echocardiography to exclude periprocedural device detachment or pericardial effusion, and accompanied by a friend or relative for the first 24 hours.

## Results

The mean age was 79 ± 8 years, with 99 of the 164 (60%) males. The left ventricular ejection fraction was preserved in 102 of the 164 (62%). Among patients who underwent mTEER, the etiology of mitral regurgitation was degenerative in 94 of the 146 (64%) and functional in 52 of the 146 (36%). MitraClip (Abbott) was implanted in 27 of the 146 (18%) and Pascal (Edwards Lifesciences) in 119 of the 146 (82%). In patients who underwent tTEER, the etiology of tricuspid regurgitation was degenerative in 2 of the 36 (5%), functional in 31 of the 36 (86%), and mixed in 3 of the 36 (8%). MitraClip was implanted in 3 of the 36 (8%), TriClip in 1 of the 36 (3%), and Pascal in 32 of the 36 (89%). The median number of devices per patient was 2 (IQR, 1-2) for mTEER and 1 (IQR 1-2) for tTEER. All cases were performed under general anesthesia with transesophageal echocardiography guidance.

A total of 26 of the 164 (16%) patients were discharged the same day as the procedure (SDD group) and 138 of the 164 (84%) were discharged after ≥1 night stay (non-SDD group). There were no significant demographic differences between these groups at baseline, and no difference with respect to etiology or severity of mitral regurgitation or tricuspid regurgitation. There was no difference between SDD and non-SDD groups in terms of procedural success (residual regurgitation grade ≥3+ following mTEER: 0/26 vs 2/138 [2%]; *P* = .548; tTEER: 0/3 vs 1/15 [7%]; *P* = .645). The median length of stay from procedure commencement was 8 hours (IQR, 7-9 hours) in the SDD group and 28 hours (IQR, 25-35 hours) in the non-SDD group (*P* < .001). The specific clinical or logistic reasons why a given patient did not undergo SDD were not available.

In order to investigate whether non-SDD patients experienced a significant delay in discharge, a further analysis was conducted to examine the number of patients discharged early (same-day and next-day) versus delayed (non-early). In total, 104 of 138 (75%) were discharged the next day. Early discharge was more commonly seen in patients having isolated mTEER (107/130 [82%] vs 21/34 [24%]; *P* = .018) compared with that in those having isolated tTEER or combined procedures. Patients in the delayed discharge group were more likely to have had an afternoon procedure (start time on or after 13:00) (56/138 [41%] vs 1/26 [4%]; *P* < .001). The proximity of the patient’s home to the hospital did not significantly differ between patients in the early and delayed discharge groups (35 [17-49] vs 31 [15-50] miles; *P* = .711).

There were no cases of periprocedural mortality or device embolization. No patients in the SDD group experienced an intraprocedural or periprocedural complication. However, complications were also rare in the non-SDD group, with no significant differences between groups (life-threatening bleeding: 0/26 vs 1/138 [1%]; *P* = .671; vascular complication: 0/26 vs 2/138 [1%]; *P* = .537; acute kidney injury: 0/26 vs 3/138 [2%]; *P* = .448; single leaflet device attachment: 0/26 vs 1/138 [1%]; *P* = .663). After a median of 6 months (IQR, 3-15 months) follow-up, all-cause mortality did not significantly differ in patients who underwent SDD versus non-SDD (1/26 [4%] vs 15/138 [11%]; *P* = .455]. Furthermore, there were no significant differences in the rates of mitral or tricuspid reintervention (1/26 [4%] vs 2/138 [1%]; *P* = .944], readmission due to heart failure (0/26 vs 13/138 [9%]; *P* = .234], or readmission for any cause (3/26 [12%] vs 13/138 [9%]; *P* = .964).

In this single-center retrospective analysis, SDD after TEER was feasible with comparable safety outcomes with non-SDD. Patients undergoing isolated tTEER, combined mitral and tricuspid TEER, or afternoon procedures were more likely to have a delayed discharge (>1 day). As TEER continues to become widely adopted, the SDD pathway is likely to be more commonly implemented. A larger prospective study is ongoing to further evaluate the safety and efficacy of the SDD approach.
